# Distribution and characteristics of rodent picornaviruses in China

**DOI:** 10.1038/srep34381

**Published:** 2016-09-29

**Authors:** Jiang Du, Liang Lu, Feng Liu, Haoxiang Su, Jie Dong, Lilian Sun, Yafang Zhu, Xianwen Ren, Fan Yang, Fei Guo, Qiyong Liu, Zhiqiang Wu, Qi Jin

**Affiliations:** 1 MOH Key Laboratory of Systems Biology of Pathogens, Institute of Pathogen Biology, Chinese Academy of Medical Sciences & Peking Union Medical College, Beijing, PR China; 2 Collaborative Innovation Center for Diagnosis and Treatment of Infectious Diseases, Hangzhou, PR China; 3 State Key Laboratory for Infectious Diseases Prevention and Control, National Institute for Communicable Disease Control and Prevention, Chinese Center for Disease Control and Prevention, Beijing, PR China

## Abstract

Rodents are important reservoir hosts of many important zoonotic viruses. The family Picornaviridae contains clinically important pathogens that infect humans and animals, and increasing numbers of rodent picornaviruses have recently been associated with zoonoses. We collected 574 pharyngeal and anal swab specimens from 287 rodents of 10 different species from eight representative regions of China from October 2013 to July 2015. Seven representative sequences identified from six rodent species were amplified as full genomes and classified into four lineages. Three lineage 1 viruses belonged to a novel genus of picornaviruses and was more closely related to Hepatovirus than to others genera of picornaviruses based on aa homology. Lineage 2, lineage 3, and lineage 4 viruses belonged to the genera Rosavirus, Hunnivirus, and Enterovirus, respectively, representing new species. According to both phylogenetic and identity analyses, Lineage 2 viruses had a close relationship with rosavirus 2 which was recovered from the feces of a child in Gambia and Lineage 3 viruses had a close relationship with domestic animal Hunnivirus. Lineage 4 viruses provide the first evidence of these enteroviruses and their evolution in rodent hosts in China.

Rodents comprise the largest group of mammals, accounting for 40% of all mammals, and comprise 35 families, 389 genera, and 2700 species^1^. As the most widely distributed group of mammals on earth, rodents come into extensive contact with humans, with opportunities to spread viruses through their urine, feces, and parasites. Rodents are important reservoir hosts of many important zoonotic viruses, including members of the Bunyaviridae, Arenaviridae, Reoviridae, Picornaviridae, and Flaviviridae^2^,^3^,^4^,^5^.

According to the International Committee on Taxonomy of Viruses (ICTV), the family Picornaviridae, order Picornavirales, currently consists of 50 species grouped into 29 existing genera and two proposed new genera. Picornaviruses are positive-sense single-stranded RNA viruses with polyadenylated genomesof 7.1–8.9 kb, composed of a single open reading frame (ORF) encoding a polyprotein with a precursor arranged in the order L, VP0, VP3, VP1, 2A, 2B, 2C, 3A, 3B, 3C, and 3D^6^,^7^. The family Picornaviridae contains clinically important human pathogens, such as *hepatovirus, poliovirus, coxsackievirus*, *rhinovirus*, and *parechovirus*, as well as several viruses that infect animals, including members of the genus *Cardiovirus*, and *Ljungan virus*^8^.

The development of metagenomic approaches and a greater focus on rodent viromes have led to increased recognition of rodent-related zoonoses caused by picornaviruses. However, the role of rodent picornaviruses in the evolution, transmission, and biology of such zoonoses in China remain unclear. We investigated the prevalence and genetic diversity of picornaviruses in rodents across mainland China by sequencing the viromes of pharyngeal and anal swabs from ten rodent species across eight provinces of China, and analyzing their phylogenetic relationships. On the basis of virome analysis using sequence-independent polymerase chain reaction (PCR) amplification, next-generation sequencing, and sequence similarity comparisons, seven representative picornavirus sequences identified from six rodent species were amplified as full genomes and classified into four lineages. Three lineage 1 viruses belonged to a novel genus of picornaviruses clustered with the genera Hepatovirus and Tremovirus. Rodent/RL/PicoV/FJ2015 virus belonged to the genus Rosavirus (rodent-related), and Rodent/Rn/PicoV/SX2015-2 clustered with the genus Hunnivirus, and had a close relationship with bovine hungarovirus. Lineage 4 viruses clustered with the genus Enterovirus, representing new species.

## Results

### Sample collection

**Figure 1:**
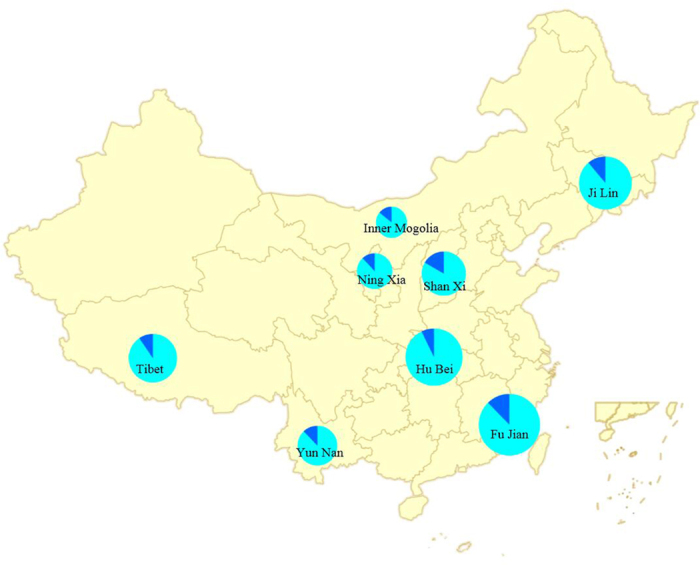
Rodent-sampling locations (eight provinces) for novel picornavirus(es) (indicated in circles) in China. The distribution map of specimens collected from eight representative regions of China was generated by online software SuperMap version 3.0 (http://www.supermapol.com/). The size of the pie chart is in proportion with the total number of specimens collected from each region. Deep blue represents samples positive for picornavirus, and light blue represents negative samples.

A total of 574 pharyngeal and anal swabs from 287 rodents of 10 different species (*Niviventer niviventer, Myodes rufocanus, Mus caroli, Dipus sagitta Pallas, Caryomys eva, Microtus clarkei, Rattus nitidus Hodgson, Cricetulus longicaudatus, Cricetulus kamensis, and Rattus losea)* were collected from eight representative regions of China (Hubei, Jilin, Yunnan, Inner Mongolia, Tibet, Ningxia, Shanxi, and Fujiang) with different altitudes and climates, from October 2013 and July 2015 (Fig. 1). Pharyngeal and anal swab specimens (1 ml each) from the same rodent species and the same site were pooled into 10 samples and processed as described previously^9^.

### Metagenomic analysis

The amplified viral nucleic acid libraries were then sequenced using an Illumina HiSeq2500 (Illumina). In total, 13,928 reads of 81-bp in length showed the best matches with Picorniviridae viral proteins in the NCBI non-redundant database. These included 3179 and 2355 reads matching hepatitis A virus (HAV) and kobuviruses, respectively, and 562, 171, and 1132 reads matching aphthoviruses, enteroviruses, and cardioviruses, respectively. The remaining 6529 sequence reads were unclassified picornavirus sequences. The presence of picornaviruses was further confirmed by PCR amplification using specific primers. Pharyngeal and anal swab specimens were positive in 37 rodents of six different species from all eight provinces (Table 1). Seven representative sequences from 37 positive samples were then amplified as full genomes.

### Genome organization

**Figure 2:**
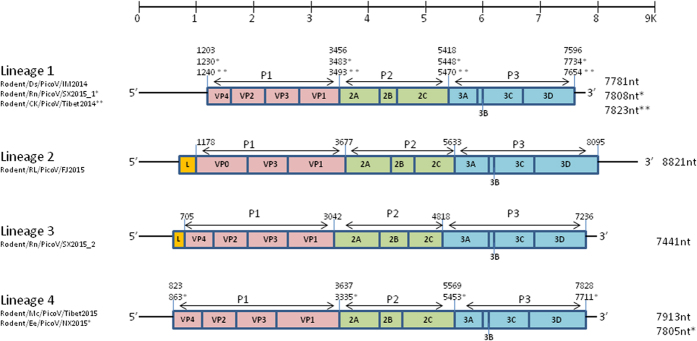
Genome organizations. Lineage 1, Rodent/RL/PicoV/FJ2015 (lineage 2), Rodent/Rn/PicoV/SX2015_2 (lineage 3), and lineage 4 rodent picornaviruses.

Seven novel rodent picornaviruses from diverse rodent species from different parts of China showed unique evolutionary characteristics and clustered into four distinct lineages. In addition to their phylogenetic clustering, the four lineages also displayed distinct genomic features, and the strains within each lineage possessed a high degree of aa and genomic feature conservation. Genome organization and size (Fig. 2), the secondary structure of the 5′UTR (Supplemental Figs 1, 2, 3, 4, 5), C + G content and ORFs (Table 2) of the seven novel rodent picornavirus were predicted. The conserved motifs KDELR, GGLPSG, YGDD, and FLKR from RNA-directed RNA polymerase were found in the 3D region of all Lineage 1, 2, 3 and 4 viurses^10^.

### Genome analyses

#### Lineage 1 viruses

The putative translation initiation site of lineage 1 viruses was contained in an optimal Kozak context (RNNAUGG) in Rodent/CK/PicoV/Tibet2014 (Tibet2014) (AAAAUGG), Rodent/Rn/PicoV/SX2015-1 (SX2015-1) (ACAAUGG), and Rodent/Ds/PicoV/IM2014 (IM2014) (GAAAUGG), respectively. The L protein was absent in lineage 1 viruses, as seen in the *enteroviruses* and *hepatoviruses*. The aa sequence identity of the P1 region between lineage 1 viruses and other picornaviruses ranged from 10.2–28.9% (Table 3), and between the three Lineage1 viruses were 75.5–90.2%. The lineage 1 viruses had protein domains characteristic of HAV protein VP1-2A, with unknown function, located in the P1 region at aa 537–681, 536–681, and 536–680, respectively. The cleavages sites of VP4/VP2 in these were not obvious in the P1 region, and were consistent as VL/GN (Table 4) in all three lineage 1 viruses. VP4 was very small (27 aa) in lineage 1 viruses, and lacked an N-terminal myristoylation signal. YPX3L “late domain” motifs (YPMYNL), which are highly conserved among primate hepatoviruses and contribute to HAV membrane envelopment by mediating capsid interactions with components of the endosomal sorting complex required for transport, were found in VP2 in all three lineage 1 viruses^11^. The P2 region of lineage 1 viruses shared 17.3–23.6% aa identity with other picornaviruses, and 76.1–86.7% with each other. The 2C protein of all of three lineage 1 viruses possessed the highly conserved GQRGSGKS (GxxGXGKS) NTP-binding site motif and the DDMGQ (DDLxQ) putative helicase-activity motif, with the third-position aa L replaced with M^12^,^13^. The P-loop domain containing nucleoside triphosphate hydrolase was located around postions 1179–1324 in the 2C protein. The P3 region in the lineage 1 viruses shared 21.8–28.4% aa identity with other picornaviruses, and 77–83.6% identity with each other. The conserved tyrosine residue at the third position of the VPg protein in all known picornaviruses was present^14^. Peptidase C3A/C3B was located in the 3C protein between aa 1528–1694 in the lineage 1 viruses. Picornavirus proteins are expressed as a single polyprotein, which is cleaved by viral C3 cysteine protease. The active-site cysteine GYCG (GxCG) was also present in the 3C protein of the three lineage 1 viruses^12^.

#### Lineage 2 viruses

The putative translation initiation site of Rodent/RL/PicoV/FJ2015 (FJ2015) is ACUAUGG, contained in an optimal Kozak context. The 5′UTR nt sequence at the positions 351–568 of FJ2015 showed 71% identity with Rosavirus 2 (489–713), which was predicted to form a type II IRES element, similar to other *rosaviruses, cardioviruses, and apthoviruses*^15^,^16^. The complete aa sequence of FJ2015 showed 51.3% and 51.4% identities with rosavirus M-7 (AEM05832) and rosavirus A2 (YP_009028557), respectively. The L protein of FJ2015 was 170 aa long, and lacked the catalytic dyad (Cys and His) of papain-like thiol proteases found in the foot-and-mouth disease virus L protein^17^. A presumed zinc-binding motif, Cys-His-Cys-Cys, found in Theiler’s murine encephalomyelitis virus and quail picornavirus^18^. L protein was also lacking. The P1 region of FJ2015 shared 47–52% aa sequence identity with rosaviruses, and 9.1–12.6% aa identity with other picornaviruses. The potential cleavage site between VP4 and VP2 was not detectable in FJ2015, as in *kobuviruses, avihepatoviruses, and parechoviruses*, and the virions probably consist of only three capsid monomers (VP0-VP3-VP1). VP1 of FJ2015 had no [PS]ALXAXETG motif. The P2 region of FJ2015 showed 43–54% aa identity with rosaviruses, and 15.7–28.3% with other picornaviruses. The 2A protein of FJ2015 contained the highly conserved picornavirus H-box/NC regions (H_994_-box/N_1056_C)^19^, while the putative 2C protease region included the NTP-binding motif (GKPGCGKS) and helicase-activity motif (DDLGQ). The P3 region of FJ2015 virus shared 60–62% aa identity with rosaviruses and 21.1–33.6% with other picornaviruses. The GXCG cysteine active site was conserved in the putative 3C protease region (GFCG). Similar to *cardioviruses and rosaviruses*, the RNA-binding domain KFRDI motif was absent from the putative 3C protease region in FJ2015.

#### Lineage 3 viruses

The almost complete 5′UTR of Rodent/Rn/PicoV/SX2015-2 (SX2015-2) showed 81% nt sequence identity with the corresponding region of OhuV-1 (HM153767). Compared with the 5′UTR of OhuV-1, the conserved core-domain motifs I-J-K-L belonging to the type II IRES was also present at nt positions 141–458 (Supplemental Fig. 1). The predicted translation initiation site of the polyprotein CAUAUGG was set in a nearly optimal Kozak context. The polypyrimidine tract was located in the 18nt upstream of the AUG initiation codon. The Yn-Xm-AUG motif of SX2015-2 was Y9-X18-AUG. The L protein of SX2015-2 was 83 aa long. The L/VP4 predicted cleavage site E/G was present in both SX2015-2 and OHuV-1, and they shared 71% aa identity in their L proteins. The L protein of SX2015-2 had no GXCG motif and no putative zinc-finger motif (C-XH-X- (5)-C-X- (2)-C)^20^,^21^. The P1 viral capsid of SX2015-2 had 85%, 84%, and 69% aa identities with BhuV-1 (JQ941880), OhuV-1, and Norway rat hunnivirus (YP009109563), respectively, and 17.4–30.1% identities with other genera of picornaviruses. The VP4 protein of SX2015-2 had a VP4 myristoylation motif GPGQSK^22^, and the predicted VP4/VP2 cleavage site was LA/DG. The P2 region of SX2015-2 (including 2A, which has high aa identity with other hunniviruses) had 68%, 70%, and 71% aa identities with BhuV-1, OhuV-1, and Norway rat hunnivirus, respectively, and 15–32.9% identity with other genera of picornaviruses. The P-loop NTPase fold was predicted in the 2C aa sequence from positions 1182–1374. The 2C protein of SX2015-2 possessed the highly conserved NTP-binding site motif GPPGQGKS and the putative helicase-activity motif DDLGQ^12^. The 3A had relatively lower aa identities of 62%, 65%, and 68% with BhuV-1, OhuV-1, and Norway rat hunnivirus, respectively, while the P3 region (excluding 3A) had higher aa identities of 90%, 88%, and 86% with BhuV-1, OhuV-1, and Norway rat hunnivirus, respectively, and 23–42.2% with other genera of picornaviruses. The conserved tyrosine residue at the third position of VPg proteins^14^. Amino acids 1600–1796 in the 3C protein were defineds cysteine peptidases belonging to the MEROPS peptidase family C3 (picornain, clan PA (C)), subfamilies C3A and C3B.

#### Lineage 4 viruses

The putative translation initiation site of lineage 4 viruses was contained in an optimal Kozak context in Rodent/Mc/PicoV/Tibet2015 (Tibet2015) (AGCAUGG) and Rodent/Ee/PicoV/NX2015 (NX2015) (GCAAUGG). The nt sequence from 295–632 of the 5′UTR of Tibet2015 had 72% identity with human coxsackievirus A11 (DQ995633), and that from 179–628 of the 5′UTR of NX2015 had 73% identity with enterovirus D68 (KT803600). Consistent with other enteroviruses, these two rodent enteroviruses had typical type I IRESs. The P1 region of lineage 4 viruses shared 16.8–32.8% aa identity with others genera of picornaviruses, and 68.6% identity with each other. Lineage 4 viruses also showed 36.3–38.2% aa identity with other members of the genus *Enterovirus*. The VP4/VP2 cleavage sites were LA/SP in the two viruses. The VP4 protein family was predicted to occur in the two lineage 4 viruses at aa positions 1–63 in Tibet2015 and 1–65 in NX2015. Interestingly, VP1 in the two novel rodent enteroviruses lacked the [PS] ALXAXETG motif present in others enteroviruses. The P2 region of the lineage 4 viruses shared 39.2–42.3% aa identity with enteroviruses, which was greater than that shared with others genera of picornaviruses (19.4–28.6%), and they also shared 70.2% aa identity with each other. The peptidase C3 domain was predicted in the 2A protein in the two lineage 4 viruses, at aa positions 815–960 and 1015–1148 of Tibet2015, and 821–961 and 1008–1133 of NX2015. The typical 2B protein, which enhances membrane permeability duration poliovirus infection, was also found in both lineage 4 viruses (Tibet2015: 1155–1317, NX2015: 1141–1278). Lineage 4 viruses also had an NTPase-motif^23^ (Tibet2015: GTPGCGKS, NX2015: GAPGTGKS) and helicase motif^13^ (Tibet2015: DDVGQ, NX2015: DDVGQ) in their 2C proteins. The P3 region of lineage 4 viruses shared 54.7–59.8% aa identity with enteroviruses, which was greater than that shared with other genera of picornaviruses (22.5–49.5%), and they shared 66% aa identity with each other. The poliovirus 3A protein-like domain was detected in both rodent enteroviruses at positions 1582–1639 and 1530–1586, respectively. This is a critical component of the poliovirus replication complex, and also inhibits transport from the host endoplasmic reticulum to the Golgi apparatus. Similar to other picornaviruses, these picornaviruses also contained the conserved GXCG motif (GQCG) in the 3C protein, which is considered to form part of the protease active site^12^. The RNA-binding domain motif KFRDI was found in both lineage 4 rodent enterovirus 3C proteins^24^.

### Phylogenetic analysis

**Figure 3:**
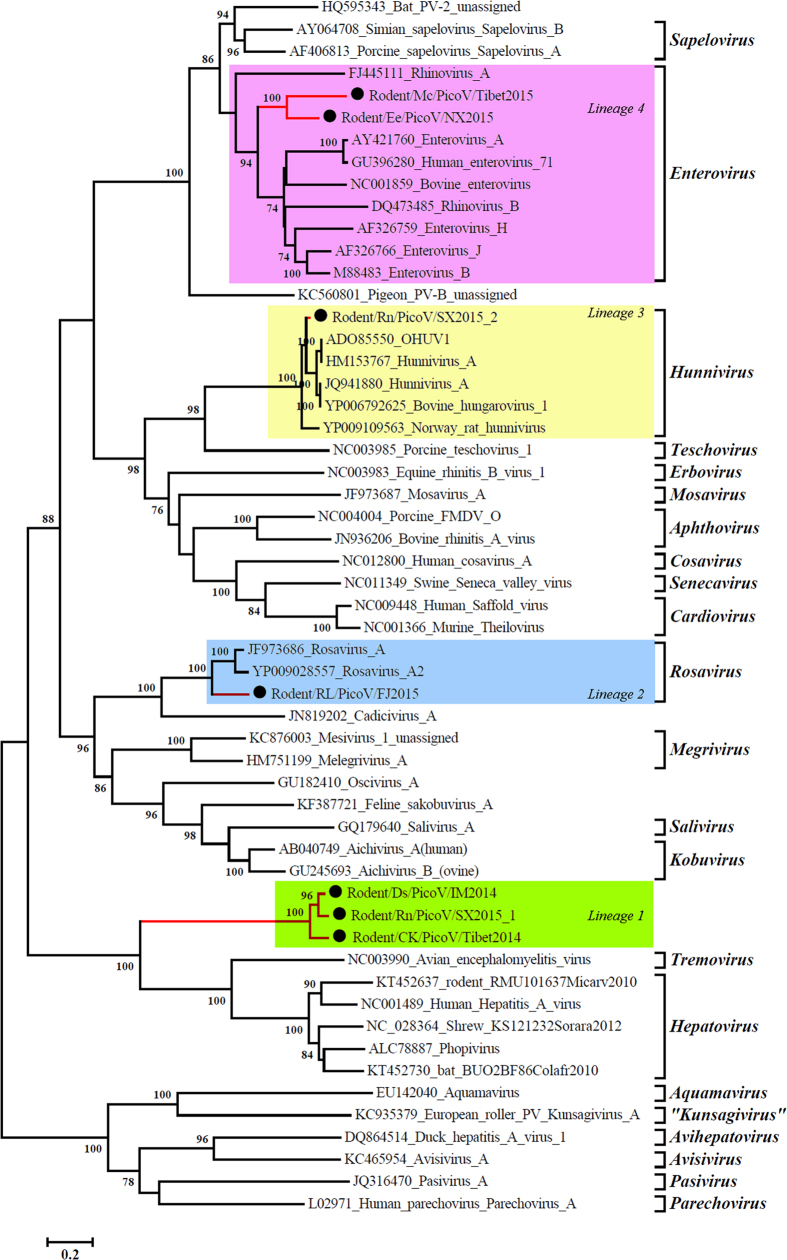
Phylogenetic tree showing the relationships between picornaviruses based on 3D polymerase amino acid sequences. Maximum likelihood mtREV with Freqs (+F) model, gamma distributed with invariant sites (G + I), with 1000 bootstrap replicates in MEGA5 (www.megasoftware.net). Proposed new genus and species names are shown on red lines and species names are prefixed by black circles (⦁).

**Figure 4:**
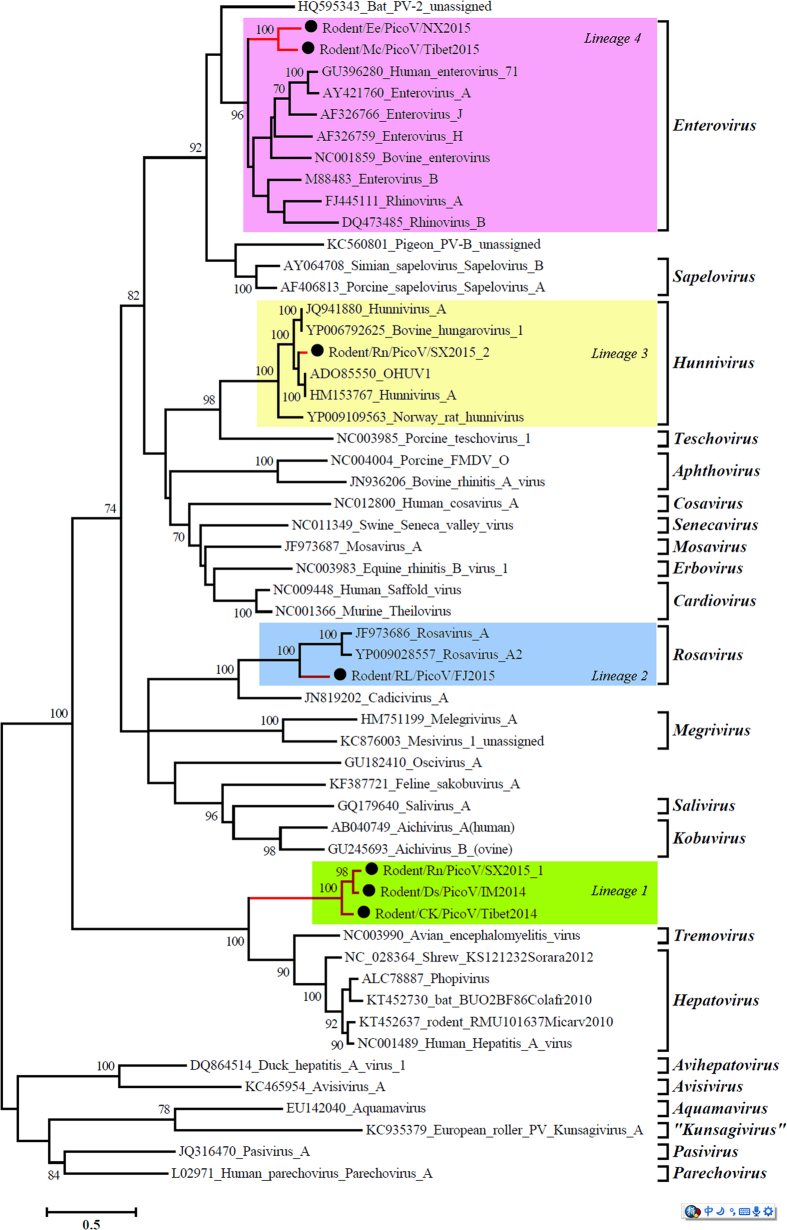
Phylogenetic tree showing the relationships between picornaviruses based on P1 region amino acid sequences. Maximum likelihood mtREV with Freqs (+F) model, gamma distributed with invariant sites (G + I), with 1000 bootstrap replicates in MEGA5 (www.megasoftware.net). Proposed new genus and species names are shown on red lines and species names are prefixed by black circles (⦁).

**Figure 5:**
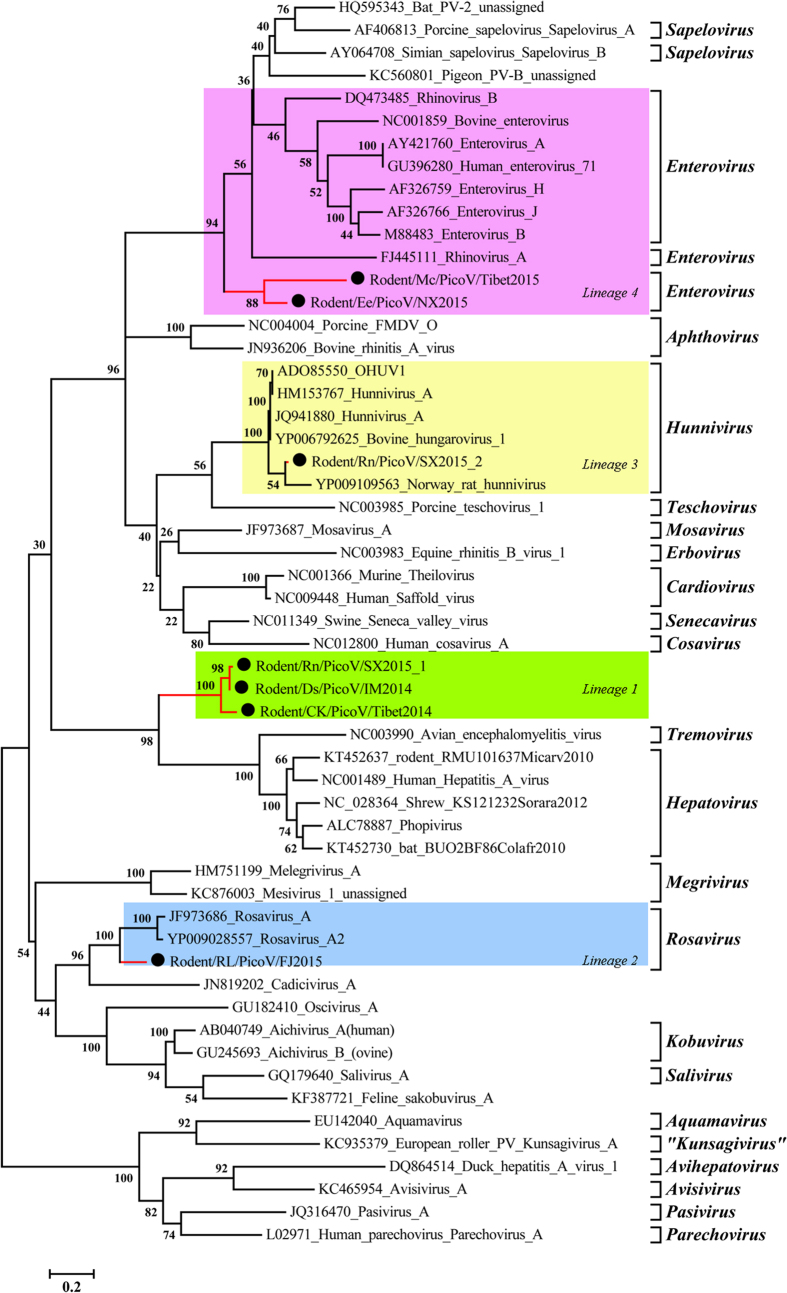
Phylogenetic tree showing the relationships between picornaviruses based on P2 region amino acid sequences (excluding 2A). Maximum likelihood mtREV with Freqs (+F) model, gamma distributed with invariant sites (G + I), with 1000 bootstrap replicates in MEGA5 (www.megasoftware.net). Proposed new genus and species names are shown on red lines and species names are prefixed by black circles (⦁).

Base on pairwise aa identities, phylogenetic analyses of the seven full genomes were conducted, which identified four distinct lineages based on 3D (Fig. 3), P1 (Fig. 4), and P2 (Fig. 5). IM2014 (*Dipus sagitta Pallas*), SX2015-1 (*Niviventer niviventer*), and Tibet2014 (*Microtus clarkei*) were clustered into lineage 1. IM2014 was most closely related to SX2015-1 for all three regions, consistent with their aa identities. They formed a unique lineage just above the genera *Tremovirus and Hepatovirus* with 96 bootstrap values, and clearly separate from other genera, suggesting that they comprise a novel genus of picornaviruses (Supplemental Fig. 6). In a phylogenetic tree of representative picornaviruses and cripaviruses based on the aa of VP2 region, lineage 1 viruses clustered with HAV to form the sole independent branch between insect and mammalian picornaviruses (Supplemental Fig. 7).

FJ2015 in lineage 2, from *Rattus losea*, together with rosavirus 2 (YP009028557) and rosavirus A (JF973686), formed a unique lineage in all three phylogenetic analyses of P1, P2, and 3D regions with 100 bootstrap values, in the genus *Rosavirus*, family Picornaviridae (Figs 3). Phylogenetic analysis of lineage 3, including SX2015-2 from Niviventer *niviventer*, indicated that it was more closely related to BhuV-1 and OhuV-1 in the 3D and P1 phylogenetic trees, consistent with the aa identities, than to the Norway rat hunnivirus within the genus *Hunnivirus*. However, the P2 phylogenetic tree placed SX2015-2 closer to the Norway rat hunnivirus than to BhuV-1 and OhuV-1. The lineage 4 rodent-associated viruses Tibet2015 and NX2015, from *Niviventer niviventer* and *Caryomys eva*, respectively, possessed higher aa identities with homologous P3 regions in others members of the genus *Enterovirus*. Phylogenetic analyses showed that these two viruses were located in the genus *Enterovirus* between rhinovirus A (FJ445111) and enterovirus A (AY421760) in terms of their 3D regions (Fig. 3), and formed an independent lineage adjacent to the other enteroviruses in phylogenetic trees based on the P1 and P2 regions (Figs 4 and 5).

## Discussion

Increasing attention focused on rodents as the natural hosts of many important zoonotic virus. Firth *et al*.^25^ identified a wide range of known and novel viruses from groups that include important human pathogens, including sapoviruses, cardioviruses, kobuviruses, parechoviruses, rotaviruses, and hepaciviruses carried by commensal *Rattus norvegicus* in New York city^25^. The role of rodent picornaviruses in the evolution, transmission, and biology of picornaviruses remains unclear. Drexler *et al*.^11^ conducted a targeted search for hepatoviruses in 15,987 specimens of 209 small mammal species, and ancestral-state reconstructions suggested a hepatovirus origin in small insectivorous mammals, and a rodent origin of human HAV^11^. Zoll *et al*.^8^ reported the first isolation, full-length sequence, characterization, and epidemiology of a Saffold virus SAFV-3 isolate^8^. Evidence suggests that this SAFV is an ubiquitous human virus causing infections early in life. It is genetically related to Theiler’s virus in rodents, and was classified as a new species in the genus Cardiovirus^8^. A novel picornavirus, provisionally named rosavirus 2, was recovered from the feces of a child in The Gambia. The complete genome of rosavirus 2 demonstrated 71.9% nt identity with its closest relative rosavirus M-7, an unclassified picornavirus identified from rodent fecal material^16^. Those findings indicated that rodents are reservoirs for a diversity of picornaviruses, and that may contribute to the disease burden of these viruses in humans. New and improved surveillance and prevention strategies for disease control should concentrate on rodent infection and disease^25^.

The seven novel rodent picornaviruses were proposed as new species or genera in the family Picornaviridae. According to the ICTV, virus taxology usually refer to their host species (or group of host species) and their defined geographic distribution. Like rodent arenaviruses and hantaviruses, a particular virus species generally only infects rodents in one subfamily or genus^26^,^27^, such as Hantaan virus in *Apodemus agrarius* and Seoul virus in *Rattus norvegicus and Rattus rattus*^28^,^29^,^30^. In contrast, the rodent picornaviruses identified in the current study showed less specificity, and a particular picornavirus species was able to infect different rodents. Lineage 1 viruses were found in *Dipus sagitta Pallas, Niviventer niviventer*, and *Microtus clarkei*, lineage 4 viruses were found in *Niviventer niviventer* and *Caryomys eva*, and FJ2015 was found in *Rattus losea*. The situation of similar strains of rodent picornaviruses occurring in different rodent genera/species is also seen in other wild or domestic animals^11^,^31^,^32^,^33^. The host range of picornaviruses appears to be reasonably broad and includes humans, canines, deer mice, bats and other rodents^1^,^34^. Rodents are the most widely distributed mammals globally and many of them live in urban or suburban environments, giving them closer human contact than most other mammals^35^,^36^,^37^. The abilities of these rodent-related picornaviruses to cross-infect other rodent species or genera leading to their spread and emergence in their closest neighbor.

HAV is an ancient and ubiquitous human pathogen, existing in both enveloped and non-enveloped forms, with a capsid structure intermediate between that of insect viruses and mammalian picornaviruses. According to the unrooted phylogenetic trees, lineage 1 viruses clustered with HAV to form the sole independent branch between insect viruses and mammalian picornaviruses. The origins of HAV were enigmatic prior to the recent discoveries of rodent-related and seal-related HAV^11^,^38^. Lineage 1 viruses possessed relatively high aa homology with hepatovirus and tremoviruses. Similar to hepatovirus, the three lineage 1 viruses lacked the L protein and possessed an HAV viral protein VP1-2A domain of unknown function, which was also found in tremoviruses and phopivirus. Non-primate hepatoviruses “late domain” (YPX3L) motifs have been identified in lineage 1 viruses, and in seal-, bat-, hedgehog-, and rodent-related hepatoviruses, but not in tremoviruses. Lineage 1 viruses still have some genomic characteristics of HAV and have only low homology with other genera of picornaviruses. Lineage 1 viruses clustered with *tremoviruses, hepatovirus* and formed an independent branch that separated them from other genera of picornaviruses near the root of the phylogenetic tree. This indicates that the *tremoviruses, hepatovirus* and lineage 1 viruses have a common ancestor and evolved in their natural hosts, independently. Their co-evolution within their respective hosts contributed to their unique genomic characteristics. HAV originates in small insectivorous mammals and has evolved for interspecies transmission to rodents, given this evolution and the contact between humans and rodents HAV eventually evolved to infect humans^11^.

BHuV-1 and OHuV-1 were isolated from cattle and sheep in Hungary in 2008 and 2009. Their complete aa sequences showed 78.8–78.9% identity with lineage 3 SX2015-2, and 72.5% identity with Norway rat hunnivirus. This indicates that SX2015-2 may be a new species in the genus *Hunnivirus*. In addition, based on the complete aa sequence identity and phylogenetic tree, our sequence was more closely related to that of domestic animal viruses(BHuV-1, OHuV-1) than to Norway rat hunnivirus, despite the distant geographic distribution and the long time intervals. This implies that picornaviruses have a worldwide geographic distribution, and a characteristic ability of cross-species transmission in humans, and wild and domestic animals^8^,^16^,^32^.

FJ2015 represents a new species of the genus *Rosavirus*, based on aa identity and phylogenetic analysis. The complete aa sequence of rosavirus2 showed 79.7% identity with rosavirusM-7 (rodent feces) and 51.4% identity with FJ2015. Our results provide the first evidence for the presence and evolution of this rosavirus in rodent hosts in China. Although there was no statistical evidence that rosavirus 2 was associated with diarrhea^16^, many rodent-borne pathogens cause only mild or asymptomatic infections in the human population, and these illnesses are often misdiagnosed and under reported^25^,^39^,^40^,^41^. It is a risk that asymptomatic infections in healthy people may evolve to become severe infectious disease in the future and in the case of the pathogens identified here this may already be occurring.

Lineage 4 viruses appeared to be new species of rodent-related enteroviruses. 3D-based phylogenetic analysis placed lineage 4 viruses adjacent to rhinovirus A and enterovirus A. Enterovirus A is the pathogen of human hand-foot-and-mouthdisease, and rhinovirus A is the human rhinovirus. These results also provide the first evidence for these enteroviruses and their evolution in rodent hosts in China, and indicate the important role of rodents in picornavirus evolution and diversity.

This study increases our understanding of picornavirus diversity in wild rodents and highlights the potentially large number of still-uncharacterized rodent picornaviruses^42^,^43^,^44^. The close relationships between picornaviruses (phylogenetic and genetic similarities) in rodents and those in domestic animals and humans have been noted previously^1^,^8^,^11^,^16^. However, further epidemiological and molecular studies are also required to investigate the geographic distribution, diversity, and clinical importance of these novel rodent picornaviruses and their hosts. A better understanding of this virus family is essential in light of its potential to contribute new human pathogens in the future^38^.

## Materials and Methods

### Ethics statement

Rodents were treated according to the Regulations for the Administration of Laboratory Animals (Decree No. 2 of the State Science and Technology Commission of the People’s Republic of China, 1988). The sampling procedure was approved by the Ethics Committee of the Institute of Pathogen Biology, Chinese Academy of Medical Sciences & Peking Union Medical College (Approval number: IPB EC20100415).

### Rodent samples

Collection of Rodent samples was conducted within the framework for hantavirus and pest monitoring activities. Capture was done using professional series live traps and conducted by the State Key Laboratory for Infectious Diseases between October 2013 and July 2015. All of the rodents were humanly euthanized. Pharyngeal and anal swabs from captured rodents were immersed in virus-sampling tubes (Yocon, China) containing maintenance medium, and temporarily stored at −20 °C. The samples were then transported to the laboratory and stored at −80 °C.

### Viral DNA and RNA library construction and next-generation sequencing

Samples from each species were pooled by combining 1 mL from each sample in maintenance medium in a fresh sample tube. The pooled samples, classified by species, were then processed using a viral particle-protected, nucleic acid purification method, as described previously^9^. The extracted RNA and DNA were amplified by sequence-independent PCR and the amplified viral nucleic acid libraries for each rodent species were sequenced using an Illumina HiSeq2500 (Illumina, San Diego, CA, USA), for single reads of 81 bp long. The raw sequence reads were then filtered using previously described criteria^9^ to obtain valid sequences.

### Taxonomic assignment

Sequence-similarity-based taxonomic assignments were done as described previously^9^. The valid sequence reads were aligned to sequences in the NCBI non-redundant nucleotide (nt) and protein databases using BLASTn and BLASTx, respectively. The taxonomies of the aligned reads with the best BLAST scores (E score <10^−5^) were parsed using the MEGAN 4 – MetaGenome Analyzer 4.

### Genome sequencing

Sequence reads classified into the same virus family or genus by MEGAN 4 were extracted. The accurate locations of the reads and the relative distances between reads of the same virus were determined based on the alignment results exported with MEGAN 4. The located reads were then used for read-based PCR to identify partial genomes. The primers used to amplify the fragments of each virus are available upon request. Based on the partial genomic sequences of the viruses, the remaining genomic sequences were determined using inverse PCR, genome walking, and 5′- and 3′-rapid amplification of cDNA end^9^.

### Distribution map

The distribution map of specimens collected from eight representative regions of China was generated by online software SuperMap (http://www.supermapol.com/). Prepared from an Excel file which contained the name of each province, the total number of samples collected and the positive samples detected. This file was then uploaded to the SuperMap online tool to generate the distribution map. The size of the pie chart is in proportion with the total number of specimens collected from each region. Deep blue represents samples positive for picornavirus, and light blue represents negative samples.

### Phylogenetic analysis

Picornaviruses containing internal ribosomal entry site (IRES)-like sequences in their 5′ untranslated regions (UTRs) were identified by BLAST searches (http://www.ncbi.nlm.nih.gov/BLAST/) of viral sequences in the GenBank database. Secondary/tertiary structural elements in picornavirus 5′UTRs were modeled using the aligned RNAalifold server of the ViennaRNA Web Services (http://rna.tbi.univie.ac.at/). MEGA5.0 (www.megasoftware.net) was used to align nt and deduced aa sequences using the MUSCLE package and default parameters. Phylogenetic trees showing the relationships among picornaviruses based on the aa sequences of P1 (capsid), P2, and P3 were generated using maximum likelihood mtREV with Freqs (+F) model, gamma distributed with invariant sites (G + I), with 1000 bootstrap replicates. Routine sequence alignments were performed using Clustal Omega, Needle (available at: http://www.ebi.ac.uk/Tools/), MegAlign (Lasergene, DNAstar, Madison, Wisconsin), and T-coffee with manual curation. The conserved protein families and domains were predicted using Pfam and InterProScan 5 (available at: http://www.ebi.ac.uk/services/proteins). Amino acid identities and genetic distances were calculated using the ML method and were performed using a Pairwise evolutionary distance (PED) calculation as the distance metric. All genome sequences have been submitted to GenBank, with accession numbers for the seven rodent picornaviruses of KX156153–KX156159.

## Additional Information

**How to cite this article**: Du, J. *et al*. Distribution and characteristics of rodent picornaviruses in China. *Sci. Rep.* **6**, 34381; doi: 10.1038/srep34381 (2016).

## Supplementary Material

Supplementary Information

## Figures and Tables

**Table 1:** Sampling locations and the total number of samples collected and rodent species.

*Scientific name*	No. of Rodents tested	No. (%) of Rodents positive for picornavirus(es)	Group (no.) of picornavirus(es) (n) detected	Sampling locations for the novel picornavirus(es) (n)
*Niviventer niviventer*	51	4		Hu Bei
*Myodes rufocanus*	48	6		Ji Lin
*Mus caroli*	29	4		Yun Nan
*Dipus sagitta Pallas*	12	2	1	Inner Mogolia
*Caryomys eva*	22	3	4	Ning Xia
*Microtus clarkei*	8	2	1	Tibet2014
*Niviventer niviventer*	30	6	3, 1	Shan Xi
*Rattus losea*	57	8	2	Fu Jian
*Niviventer niviventer*	30	2	4	Tibet2015

**Table 2:** Genomic features of rodent picornaviruses.

**Name**	**Size (bp)**	**C + G(%)**	**coding region**	**polyprotein (amino acids)**	**5′URT (nucleic acid)**
Rodent/Ds/PicoV/IM2014	7781 nt	38.53	1203–7595	2131	1202
Rodent/Rn/PicoV/SX2015-1	7808 nt	41.07	1230–7736	2169	1229
Rodent/CK/PicoV/Tibet2014	7823 nt	39.47	1240–7653	2138	1239
Rodent/RL/PicoV/FJ2015	8821 nt	50.84	668–8095	2476	668
Rodent/Rn/PicoV/SX2015-2	7441 nt	45.48	459–7238	2260	458
Rodent/Ee/PicoV/NX2015	7805 nt	46.09	863–7711	2283	862
Rodent/Mc/PicoV/Tibet2015	7913 nt	50.73	823–7830	2335	822

**Table 3:** Amino acid identity of rodents picornaviruses and representatives of other genera.

Genus	picornavirus		Lineage 1			Lineage 2			Lineage 3			Lineage 4		
	Name	Accession no	P1	P2^a^	P3^c^	P1	P2^a^	P3^c^	P1	P2^a^	P3^c^	P1	P2^a^	P3^c^
Aphthovirus	Foot and mouth disease virus O	NC004004	11.2–14.4	18.2–18.7	26.3–27.1	10.5	20.3	28.5	20.6	27.5	33.5	16.8–17.7	25.1–25.9	29.1–29.3
Avihepatovirus	Duck_hepatitis_A_virus_1	DQ864514	18.1–19	18–18.8	27.3–27.9	12.3	15.7	24.2	17.9	17.8	24.5	16.8–18.4	19.4–20.5	24.2–25.1
Cardiovirus	Human_Saffold_virus	NC009448	15.1–16.2	18.7–19.6	23.5–24.3	11.8	18	29	25.3	22	34.1	23.3–23.6	24.4–25.1	24.1–25.2
	Murine_Theilovirus	NC001366	15.3 ~ 16.1	19.1–19.3	24.2–24.7	12.3	17.7	27.6	26	21.7	35.4	22.3–22.5	22.1–22.3	25.3–26.1
Enterovirus	Human_enterovirus_71	GU396280	14.3–15.1	18.6–18.8	27.3–28.4	11.4	19.1	28	22.4	19.7	36.6	37.1–38.2	39.2–39.5	54.7–54.9
	Enterovirus_J	AF326766	15.2–16.3	18.2–18.6	26.7–27.5	11.2	19.6	27.2	22.2	19.9	37.9	36.3–37	40–42.3	58.6–59.8
Erbovirus	Equine_rhinitis_B_virus_1	NC003983	13–13.6	18.6–19.1	24–24.2	10.9	17.7	26.9	23.9	25.1	31.6	23.1–23.2	22.8–23.3	28–29.6
Hepatovirus	Hepatitis_A_virus	M14707	27.1–28.1	23.2–23.3	23.9–24.9	10.1	17.2	25.1	19.9	16.7	24.6	19–20.6	20.6–20.8	26.9–27.2
	rodent_RMU101637Micarv2010	KT452637	27.2–28.9	19.4–20.7	27.4–27.5	10.1	17.7	26.4	20.2	17.1	25.8	18.1–20.4	21.9–22.4	24.8
Kobuvirus	Aichivirus_A(human)	AB040749	12.6–13	18.8–18.9	25.1–25.4	11.2	28.3	32.1	19.5	19.3	28.2	19.5–22.5	23.4–23.8	25.5–25.9
	Aichivirus_C_(porcine)	EU787450	13.9–14.2	18–18.3	24.6–24.2	10.8	27.3	33.6	19.8	19	28.8	21.5–23.9	21.5–22.2	26.7–27.7
Parechovirus	Human_parechovirus_ParechovirusA	L02971	17.2–18.3	18.1–18.4	25.9–26.2	9.1	17	21.1	18.2	18.1	23	19.3–21.8	19.6–20.7	22.5–23.7
Senecavirus	Swine_Seneca_valley_virus	NC011349	13.4–14.4	18.6–19.9	22.5–22.8	11.5	19.1	27.9	26.2	24.9	35	23.5–25.3	24.9–25.1	24.3–25.6
Teschovirus	Porcine_teschovirus_1	NC003985	13.2–13.5	18.1–18.7	21.9–23	11.7	19.2	29.5	30.1	32.9	42.8	22.5–22.6	25.6–26.7	28.2–28.3
Tremovirus	Avian_encephalomyelitis_virus	NC003990	27.1–28.1	17.4–18.9	24.3–25.8	10.1	17.1	25.6	21.4	18.7	25.8	17.9–18.4	20–20.1	25.5–26.6
Sapelovirus	Simian_sapelovirus_Sapelovirus_B	AY064708	12.4–12.7	22.7–23.9	25.3–25.4	11.9	17.8	28.2	24.3	20.3	29.4	31.5–32	27.7–28.6	49.1–49.5
	Avian_sapelovirus	AY563023	11.9–12.2	20–20.4	25.6–26.4	10	19	27.2	22.8	22.1	30.5	25.1–26.8	26.8–27	46.5–47.9
Rosavirus	Rosavirus_A	JF973686	14.6–16	19.2–19.7	21.8–22.3	31.8	51.7	64.7	19.4	20.1	29.7	21–21.2	20.8–20.9	27.3–28
Cosavirus	Human_cosavirus_A	NC012800	12.7–15.1	19.8–20.2	23–23.6	12.6	20.4	28.5	25.3	15	34.8	24.3–25.4	22.7–24.3	27.4–28.4
Mosavirus	Mosavirus_A	JF973687	11.9–13.4	19.8–20.5	23.7–24.5	11.3	21.7	30.7	24	16.3	31.7	23.4–23.5	26.6–28.6	26.9–28.1
Hunnivirus	Hunnivirus_A	JQ941880	12.1–14.7	19.4–20.2	23.5–23.9	11.2	19.7	31.6	72.6	68	90.3	23.6–24.2	20.9–21	28.6–29.1
Megrivirus	Mesivirus_1	KC876003	10.2–12.4	19.5–20.2	21.8—22.5	9.8	27.1	28.7	17.4	18.8	32.5	20.8–21	21.7–22.4	26.4–27.2
Unclassified	Bat_PV-2	HQ595342	12.8–14.3	17.5–17.8	25.1–25.8	11.8	19.5	29.7	22.6	19.4	29.1	31.5–32.2	27–28.3	47.3–49
Unclassified	BtNv-PicoV/SC2013	KJ641697	15.7–16.2	17.7–18.5	23.9–24.5	11.9	19	28.8	25.2	19.9	29.6	30.1–32.8	27.8–28.1	46.7–47.2
Unclassified	Pigeon_PV-B_unassigned	KC560801	13.6–15.7	19.4–20.1	24.4–24.8	10.8	18.5	31.1	23.7	21.7	28.4	28.2–29.5	22.9–24.6	39.7–40.6

^a^Shown is a comparison of genomic features of group 1, 2, and 3 bat picornaviruses, representative species of other genera, and amino acid identities between the predicted P1, P2 (excluding 2A), P3 (excluding 3A), 3Cpro, and 3Dpol proteins of bat picornavirus groups 1, 2, and 3 and the corresponding proteins of representative species of other picornavirus genera. b P2 region excluding 2A. c P3 region excluding 3A.

**Table 4:** Protease-cleavage sites of Group 1–6 viruses.

Cleavage between	Lineage 1			Lineage 2	Lineage 3	Lineage 4	
	Rodent/Ds/PicoV/IM2014	Rodent/Rn/PicoV/SX2015-1	Rodent/CK/PicoV/Tibet2014	Rodent/RL/PicoV/FJ2015	Rodent/Rn/PicoV/SX2015-2	Rodent/Ee/PicoV/NX2015	Rodent/Mc/PicoV/Tibet2015
L/VP4					FE/GP		
L/VP0				AQ/RH			
VP4/VP2	VL/GN	VL/GN	VL/GN		LA/DG	LA/SP	LA/SP
VP0/VP3				VQ/ST			
VP2/VP3	LQ/GL	LQ/GL	LQ/GL		FE/GL	TQ/GI	TQ/GL
VP3/VP1	FQ/GD	FQ/GD	FQ/GD	SQ/LT	LQ/GE	LQ/GP	KT/GV
VP1/2A	NQ/AV	NQ/AV	NQ/SA	QQ/VS	PA/SR	GQ/EG	WL/GD
2A/2B	NG/ID	NG/ID	NG/VD	TS/GY	PG/PF	EQ/GL	QQ/GC
2B/2C	CQ/GK	TQ/AE	CQ/SD	EK/GD	FE/GP	KQ/GD	KQ/GD
2C/3A	MQ/AL	MQ/AL	MQ/AL	EN/SE	FE/GP	LQ/GS	LQ/GP
3A/3B	YQ/GP	YQ/GP	YQ/GP	AE/GA	ME/GA	IQ/GP	LQ/GP
3B/3C	AN/PK	PK/PS	EQ/PR	FE/GL	FQ/GP	IQ/GP	IQ/GP
3C/3D	PN/RM	PS/RM	PA/RL	CE/GL	FQ/GR	EQ/GQ	HQ/GQ

*Determined by The multiple alignments with other relevant picornavirus and predicted using Pfam and InterProScan 5.
