# Supplementary material for: Distribution and characteristics of rodent picornaviruses in China
Source: Sci Rep. 2016 Sep 29;6:34381. doi: 10.1038/srep34381 (PMC5041129; doi:10.1038/srep34381)
Supplement: Supplementary Information [file srep34381-s1.pdf]

Distribution and characteristics of rodent picornaviruses in China

Jiang Du[1], Liang Lu[3], Feng Liu[1], Haoxiang Su[1], Jie Dong[1], Lilian Sun[1], Yafang Zhu[1], Xianwen Ren[1], Fan Yang[1], Fei Guo[1]*, Qiyong Liu[3]*, Zhiqiang Wu[1,2,]*, Qi Jin[1,2]*

[1]MOH Key Laboratory of Systems Biology of Pathogens, Institute of Pathogen Biology, Chinese Academy of Medical Sciences & Peking Union Medical College, Beijing, PR China

[2]Collaborative Innovation Center for Diagnosis and Treatment of Infectious Diseases, Hangzhou, PR China

[3]State Key Laboratory for Infectious Diseases Prevention and Control, National Institute for Communicable Disease Control and Prevention, Chinese Center for Disease Control and Prevention, Beijing, PR China

*****These authors contributed equally to this work and are co-senior authors.

Corresponding authors:E-mail addresses: zdsys@vip.sina.com (Qi Jin); wuzq2009@ipbcams.ac.cn (Zhiqiang Wu); liuqiyong@icdc.cn (Qiyong Liu);

Supplemental Figure 1.

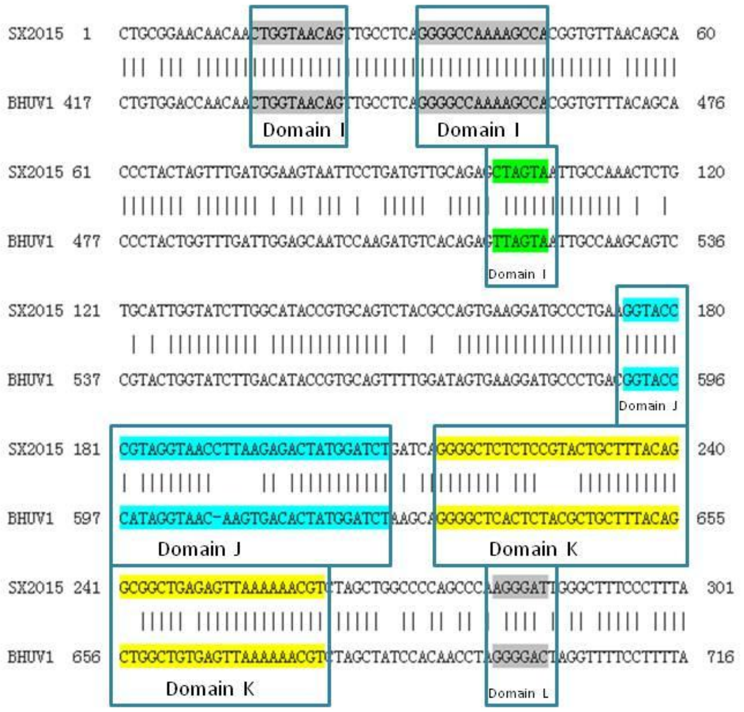

5′ UTRs of Rodent/Rn/PicoV/SX2015-2and OHUV1/2009/HUN. The conserved motifs of the core domains I-J-K-L represent the type II internal ribosomal entry site atnt positions 141–458.

Supplemental Figure2.

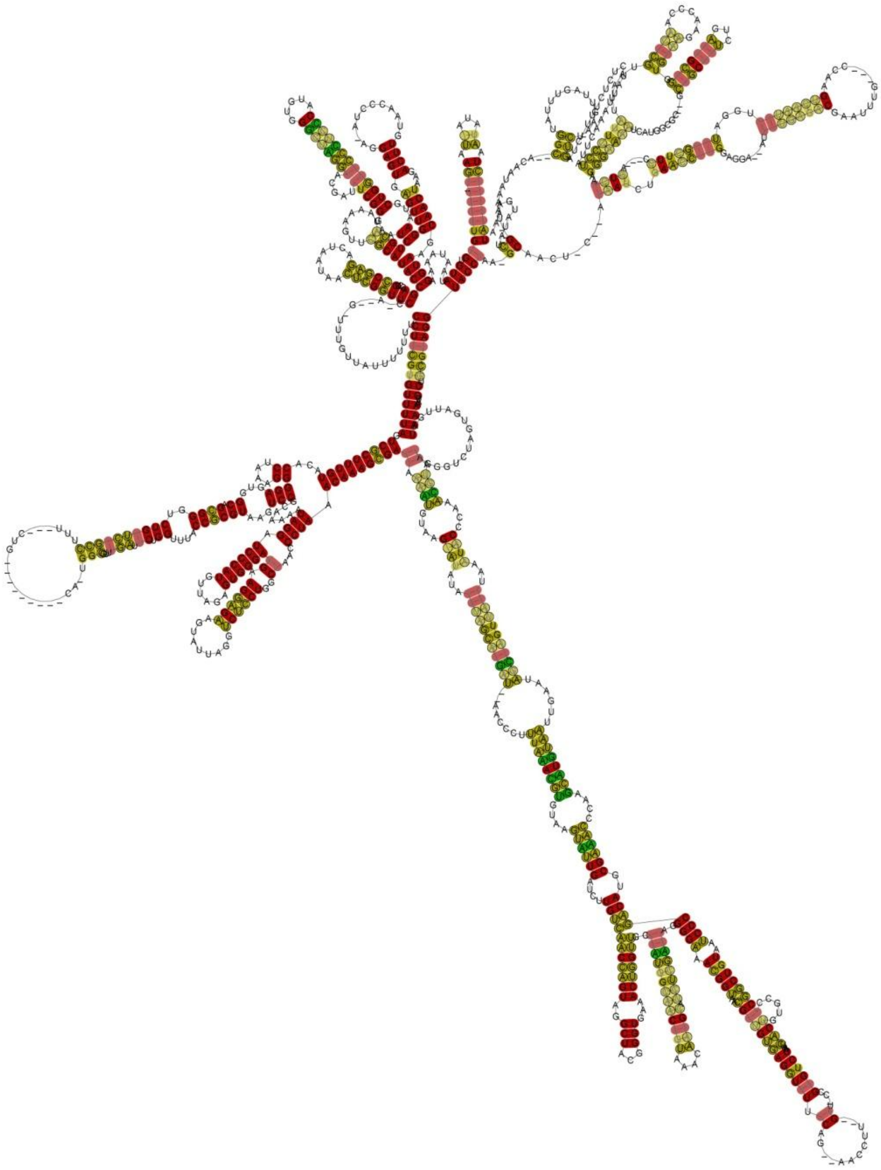

Secondary/tertiary structural elements in lineage 1 virus 5′ UTRs were modeled using the aligned RNAalifold server of the ViennaRNA Web Services(http://rna.tbi.univie.ac.at/).

Supplemental Figure 3.

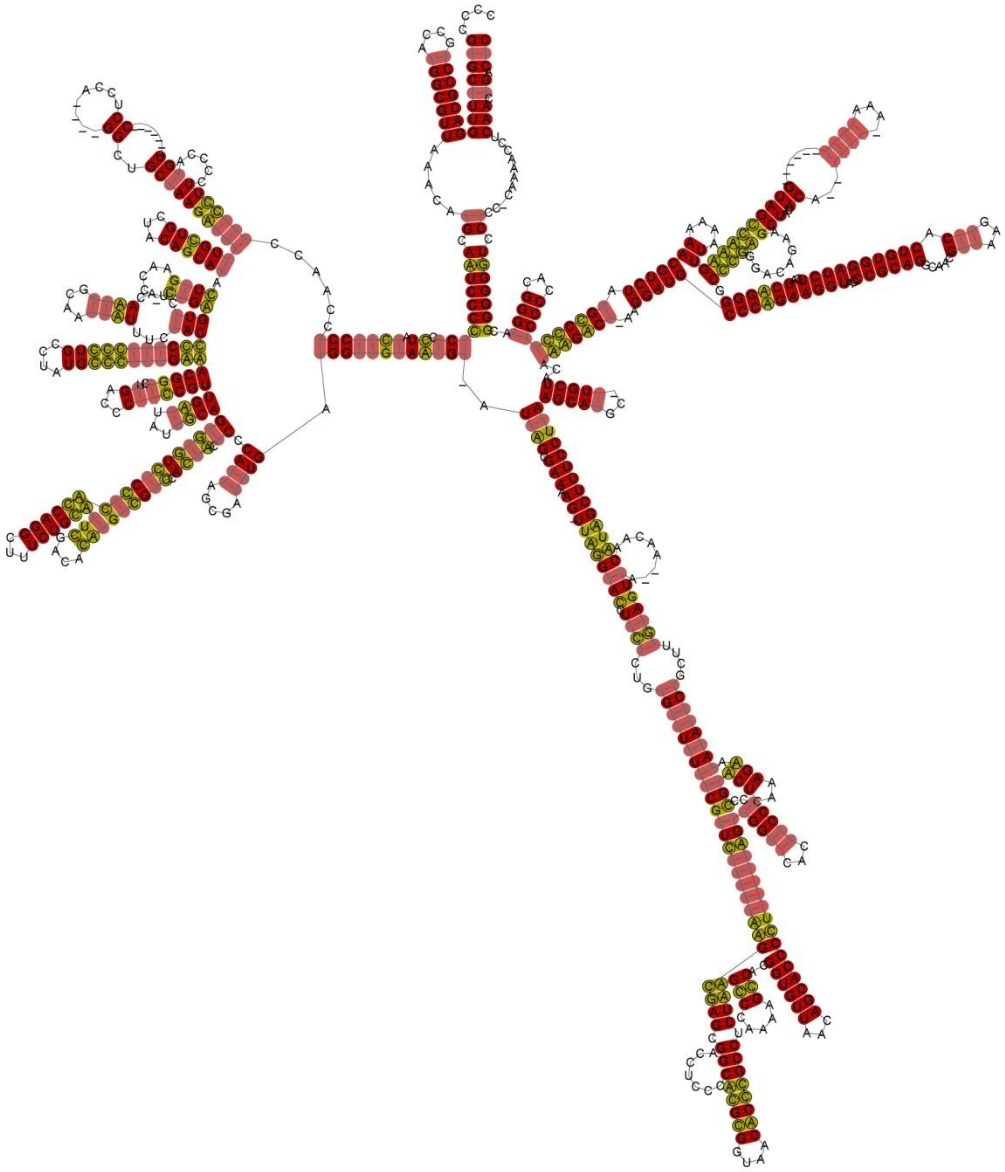

Secondary/tertiary structural elements in Rodent/RL/PicoV/FJ2015 5′ UTRs were modeled using the aligned RNAalifold server of the ViennaRNA Web Services(http://rna.tbi.univie.ac.at/).

Supplemental Figure 4.

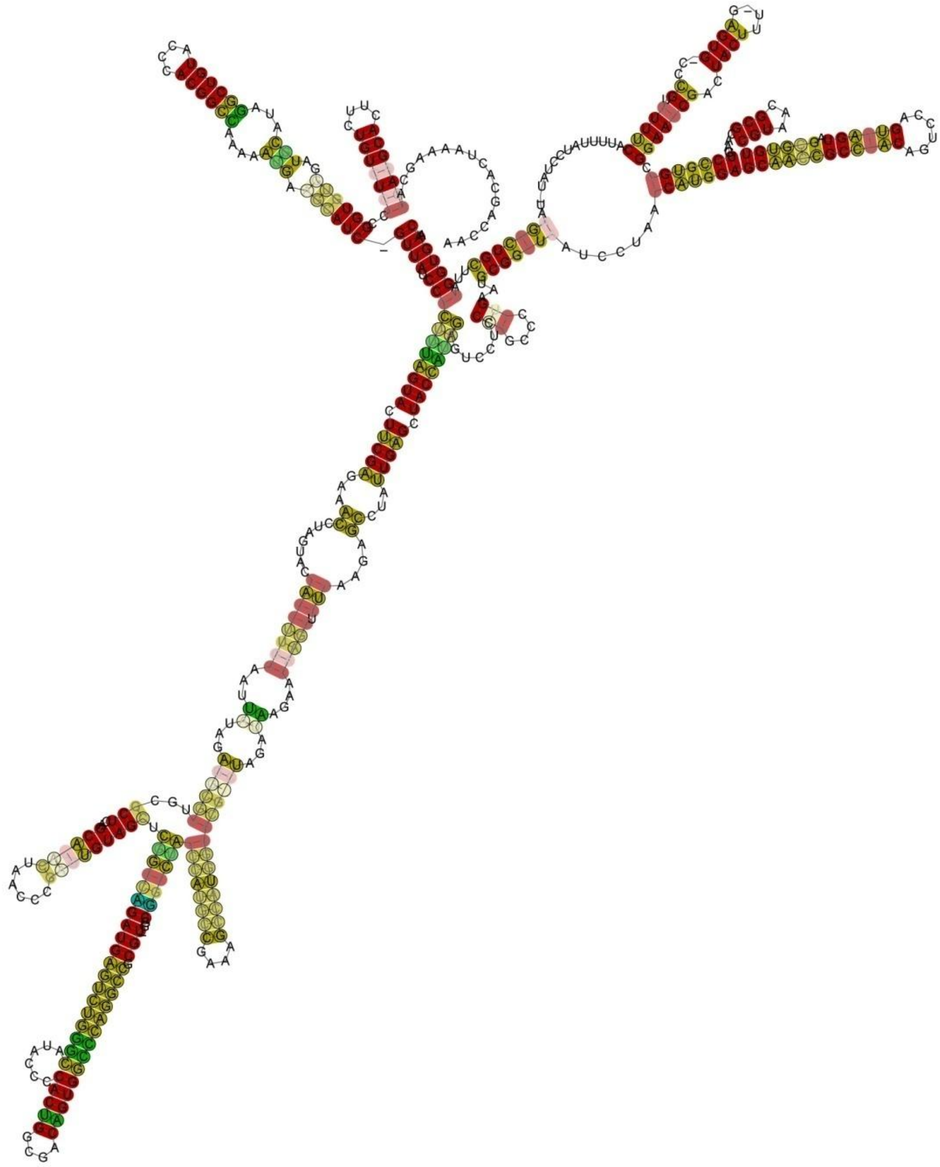

Secondary/tertiary structural elements in Rodent/Rn/PicoV/SX2015_2 5′ UTRs were modeled using the aligned RNAalifold server of the ViennaRNA Web Services(http://rna.tbi.univie.ac.at/).

Supplemental Figure 5.

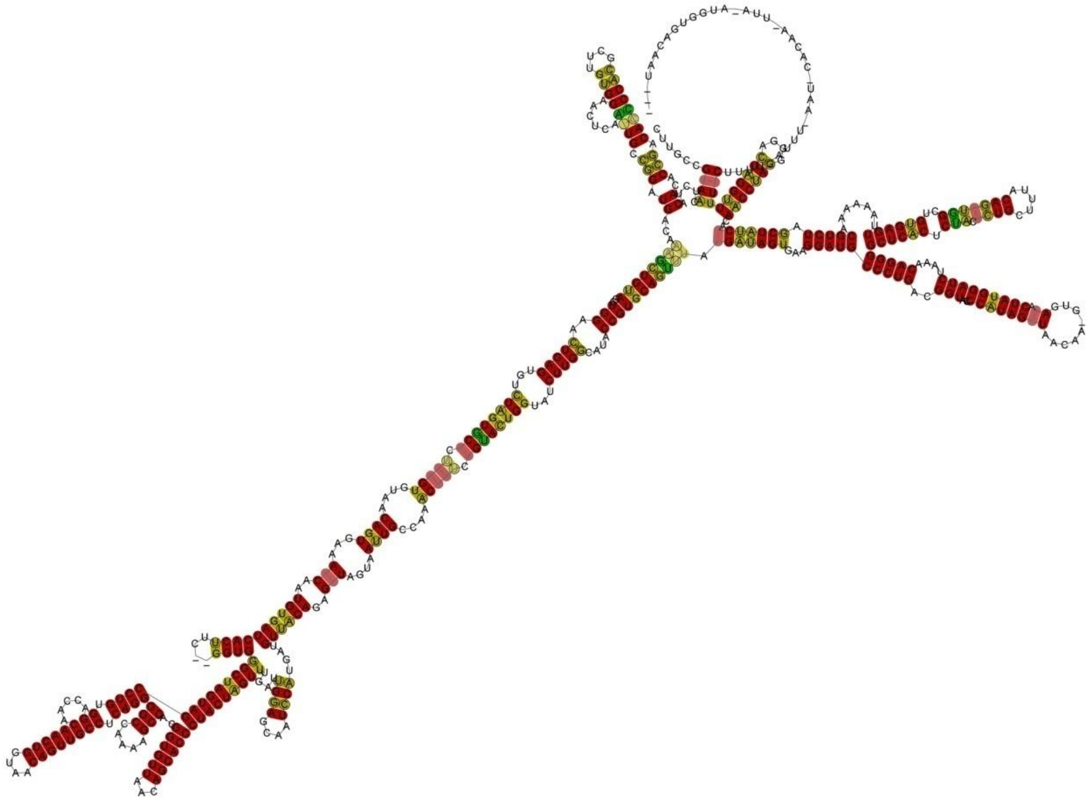

Secondary/tertiary structural elements in lineage 4 virus 5′ UTRs were modeled using the aligned RNAalifold server of the ViennaRNA Web Services(http://rna.tbi.univie.ac.at/).

Supplemental Figure 6.

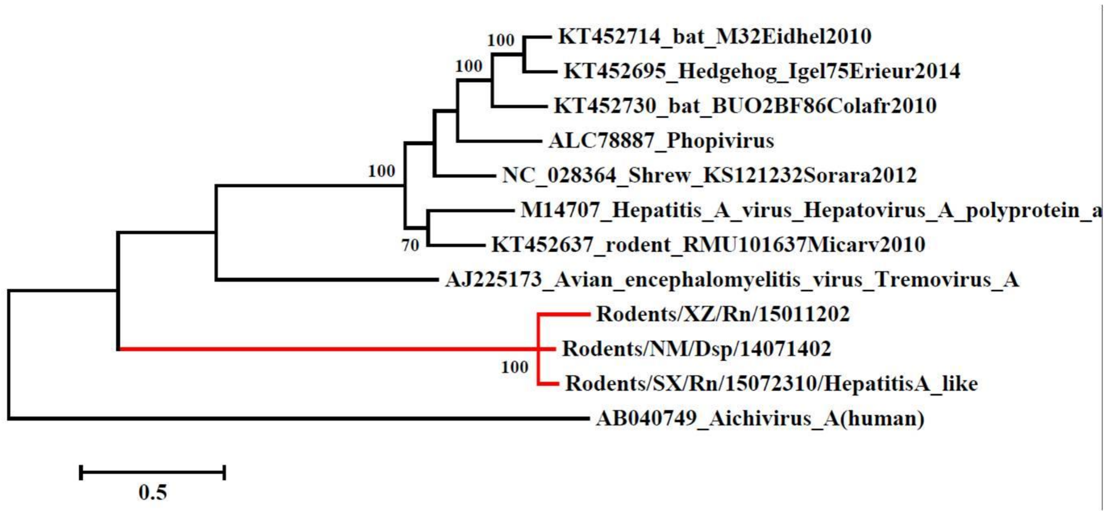

Phylogenetic tree showing the relationships between lineage 1 virusesand hepatovirus and tremoviruses based on the amino acid sequences of 3D polymerase, constructed using maximum likelihood mtREV with Freqs (+F) model, gamma distributed with invariant sites (G+I), with 1000 bootstrap replicates in MEGA5 (www.megasoftware.net). Proposed new genus and species are shown along the red line.

Supplemental Figure 7

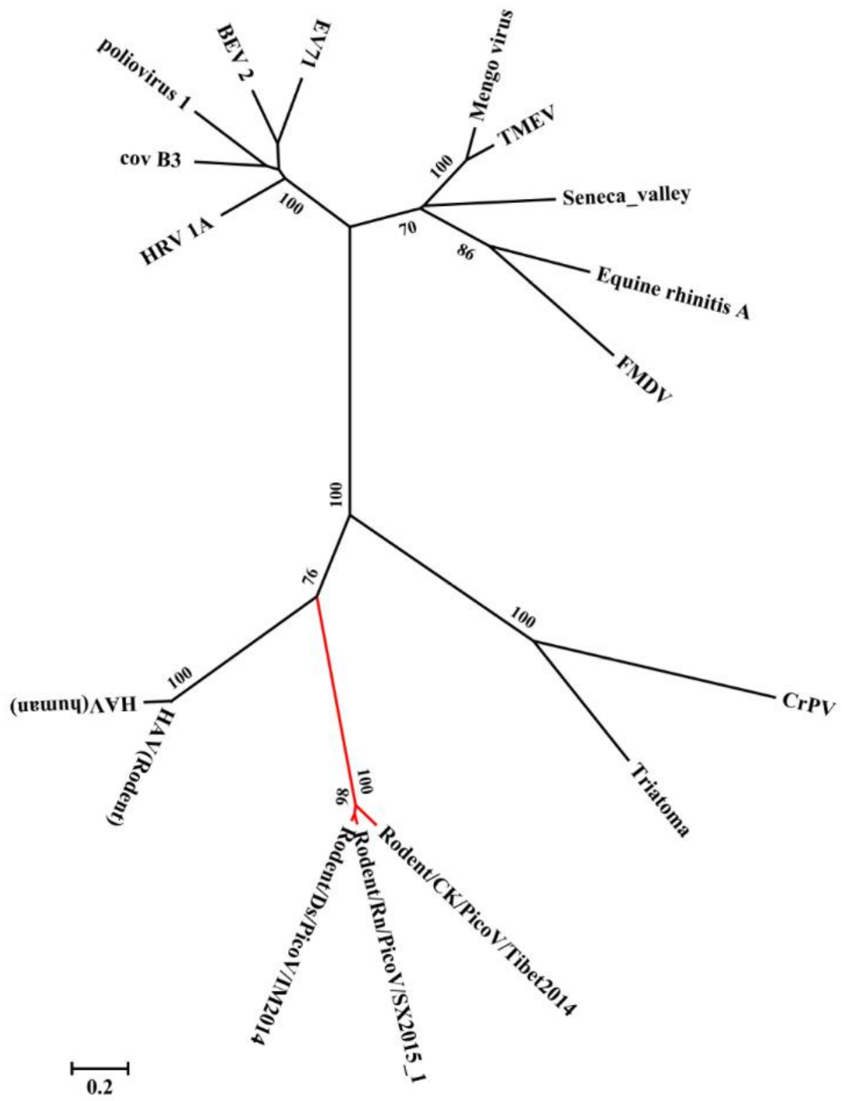

Phylogenetic unrooted tree with representativepicornaviruses,lineage 1,and cripaviruses, based on the VP2regionamino acid sequence. 1COV (coxsackievirus B3), 1TME (Theilers virus),2MEV (Mengo virus), 3CJI (Seneca Valley virus), 1ZBA (FMDV A10), 2WFF (equine rhinitis A virus), PDB accessions 3VBF (EV71), 1BEV (bovineenterovirus), 4HRV (human rhinovirus14), 1HXS (poliovirus type1), 3NAP (Triatoma virus), and 1B35 (CrPV).
